# Emerging trends and hotspot in gut–lung axis research from 2011 to 2021: a bibliometrics analysis

**DOI:** 10.1186/s12938-022-00987-8

**Published:** 2022-04-21

**Authors:** Zhendong Wang, Chen Bai, Tingyao Hu, Changyong Luo, He Yu, Xueyan Ma, Tiegang Liu, Xiaohong Gu

**Affiliations:** 1 School of Traditional Chinese Medicine, Beijing University of Chinese Medicine, Beijing, 100029 China; 2 School of Acupuncture-Moxibustion and Tuina, Beijing University of Chinese Medicine, Beijing, 100029 China; 3 Department of Infectious Diseases, Dongfang Hospital Beijing University of Chinese Medicine, Beijing, 100078 China

**Keywords:** Bibliometric, Knowledge map, Gut–lung axis, Inflammation

## Abstract

**Background:**

Increasing attention has been paid to the potential relationship between gut and lung. The bacterial dysbiosis in respiratory tract and intestinal tract is related to inflammatory response and the progress of lung diseases, and the pulmonary diseases could be improved by regulating the intestinal microbiome. This study aims to generate the knowledge map to identify major the research hotspots and frontier areas in the field of gut–lung axis.

**Materials and methods:**

Publications related to the gut–lung axis from 2011 to 2021 were identified from the Web of Science Core Collection. CiteSpace 5.7.R2 software was used to analyze the publication years, journals, countries, institutions, and authors. Reference co-citation network has been plotted, and the keywords were used to analyze the research hotspots and trends.

**Results:**

A total of 3315 publications were retrieved and the number of publications per year increased over time. Our results showed that *Plos One* (91 articles) was the most active journal and The United States (1035 articles) published the most articles. We also observed the leading institution was the University of Michigan (48 articles) and Huffnagle Gary B, Dickson Robert P and Hansbro Philip M, who have made outstanding contributions in this field.

**Conclusion:**

The *Inflammation*, *Infection* and *Disease* were the hotspots, and the regulation of intestinal flora to improve the efficacy of immunotherapy in lung cancer was the research frontier. The research has implications for researchers engaged in gut–lung axis and its associated fields.

## Introduction

With the development of microbial analysis technology and bioinformatics, microbial research has greatly expanded its scope. The gut, a critical immune organ, harbors a flora of microorganisms [1]. Increasingly, there is a mounting evidence to suggest the regulation of intestinal flora and its metabolites on distal organs, which in turn affects the occurrence and development of diseases [2–4]. Concepts such as “gut–brain axis”, “gut–liver axis”, “gut–lung axis” were developed to illustrate the relationship between organs. The gut–lung axis is bidirectional and is the crosstalk between the respiratory and digestive system [5]. According to the theories of traditional Chinese medicine, lung and intestines are in a closely related organ system [6]. The intestines and lung are homologous structurally from a histological embryological point of view [7]. Studies have found the destruction of intestinal integrity in patients with chronic obstructive pulmonary disease (COPD) [8]. Patients with lung cancer have gastrointestinal dysmotility as well [9]. Studies have shown that respiratory symptoms and pulmonary function changed in patients with intestinal bowel disease (IBD) and intestinal bowel syndrome (IBS) even without acute or chronic respiratory diseases [10, 11]. Gastroesophageal reflux disease would cause respiratory symptoms and aggravate the existing respiratory diseases [12]. Researchers have also found that lipopolysaccharide (LPS) atomization and high-calorie diet synergistically promoted the pulmonary inflammatory process in rat, that is relevant to the change in gut microbiota [13]. The above studies have confirmed the close relationship between intestines and lung, especially in the pathological states involving inflammation.

Bibliometrics is used to evaluate the information of literature, and the database Web of Science Core Collection (WoSCC) is often used in the bibliometric analysis. CiteSpace, a software developed by Chaomei Chen [14], has become a key tool for bibliometric analysis in recent years. It is applied to generate visual knowledge map to explore the knowledge domain [15]. Based on WoSCC, Xiaoquan Huang performed a global bibliometric analysis from 1998 to 2018 and evaluated the emerging trends in the field of gastrointestinal microbiology, whose study has found that the new therapeutic targets in intestinal microflora would be the focus of future research [16]. However, there is no bibliometric analysis in the field of the gut–lung axis.

To analyze the research situation and trends concerning the gut–lung axis within the past 10 years, CiteSpace 5.7.R2 was applied in this study, which aims to identify the key authors, institutions, countries, important journals, research focuses and emerging trends in this field.

## Results

### Distribution of articles by publication years

**Fig. 1:**
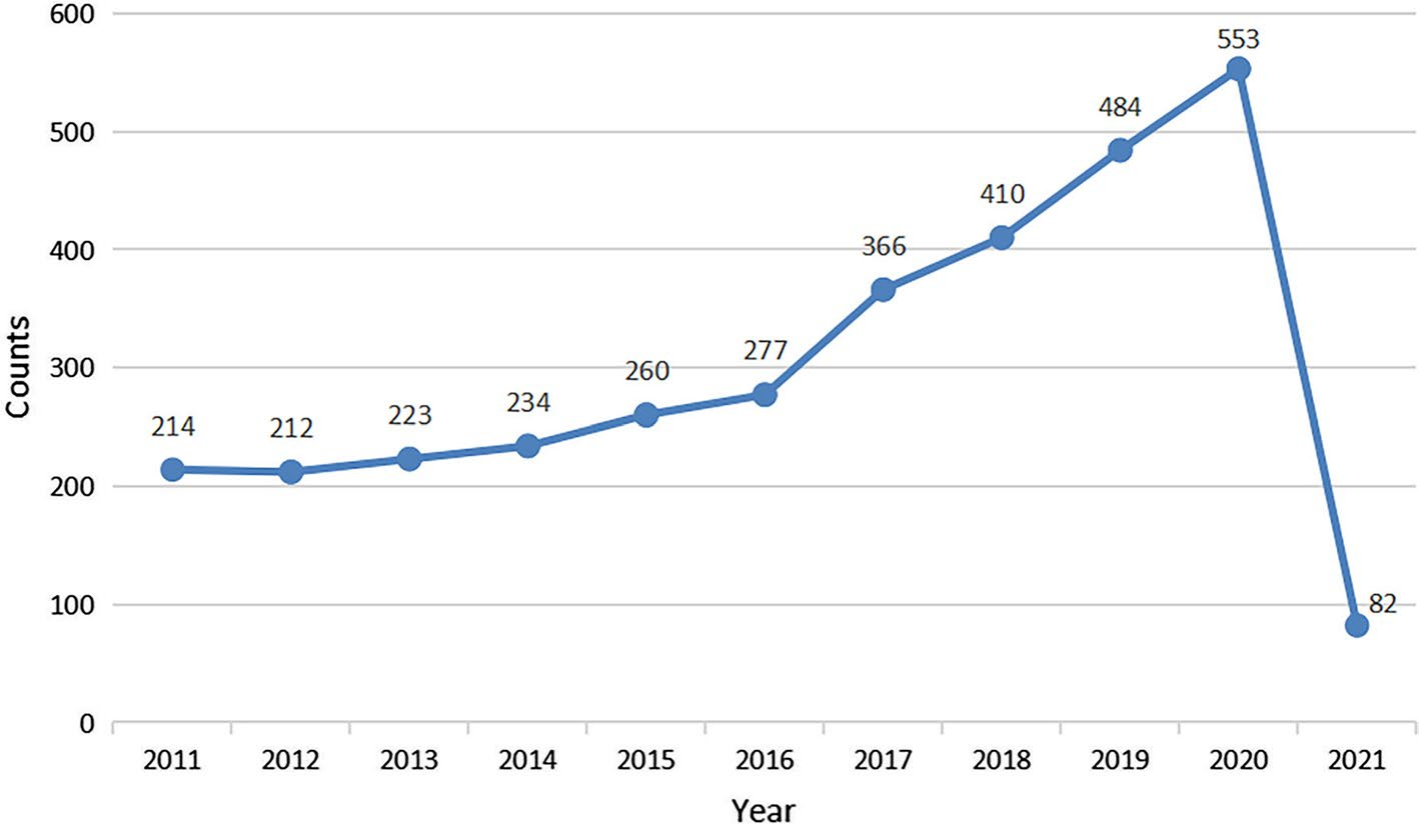
Trend of publications in the field of gut–lung axis from 2011 to 2021

From 2011 to 2021, 3,315 articles were published. There was an increasing trend for a quantity of research publications on gut–lung axis, from 214 in 2011 to 553 in 2020 (Fig. 1), which indicates an increasing interest in this field in recent years.

### Funding source

The top 10 major funding sources are shown in Table 1. United States Department of Health and Human Services, National Institutes of Health, National Natural Science Foundation of China mainly funded this field. The United States and China contributed the most fundings in this field.

**Table 1:** Top 10 funding sources

Ranking	Funding source	Country/region	Frequency
1	United States Department of Health and Human Services	United States	591
2	National Institutes of Health	United States	588
3	National Natural Science Foundation of China	China	300
4	European Commission	Europe	186
5	National Heart, Lung, and Blood Institute	United States	159
6	National Institute of Allergy and Infectious Disease	United States	152
7	National Cancer Institution	United States	129
8	National Institute of Diabetes and Digestive and Kidney Diseases	United States	103
9	Ministry of Education, Culture, Sports, Science and Technology	Japan	89
10	Japan Society for the Promotion of Science	Japan	84

### Journal analysis

The top 10 journals are listed by the number of publication in Table 2, which have published 361 articles in total and accounts for about 11% of the total number of publications. *Plos One* has published 91 articles, followed by *Frontiers in Immunology*, 73 articles. The impact factor (IF) of the 10 journals ranged from 2.192 to 7.561. The top 10 cited journals are listed in Table 3. *Plos One* was the most active journal (1586 citations), followed by *Proceedings of the National Academy of Sciences of the United States of America* (1,409 citations). In addition, articles in top journals such as *New England Journal of Medicine*, *Lancet*, *Nature* and *Science* were widely cited in the field of the gut–lung axis.

**Table 2:** Top 10 most publication journals

Ranking	Journal	Frequency	IF
1	*Plos One*	91	3.24
2	*Frontiers in Immunology*	73	7.561
3	*Scientific Reports*	45	4.379
4	*Frontiers in Microbiology*	36	5.640
5	*International Journal of Molecular Sciences*	25	5.923
6	*Oncotarget*	20	5.168*
7	*Mucosal Immunology*	19	7.313
8	*World Journal of Gastroenterology*	18	5.742
9	*Journal of Immunology*	17	5.422
10	*Journal of Surgical Research*	17	2.192

IF, impact factors in 2020. *, the impact factors of *Oncotarget* in 2016.

**Table 3:** Top 10 most cited journals

Ranking	Journals	Citation times	IF
1	*Plos One*	1586	3.24
2	*Proceedings of the National Academy of Sciences of the United States of America*	1409	11.205
3	*Nature*	1364	49.962
4	*Science*	1184	47.728
5	*New England Journal of Medicine*	1044	91.245
6	*Cell*	890	41.582
7	*American Journal of Respiratory and Critical Care Medicine*	878	21.405
8	*Nature Medicine*	848	53.44
9	*Journal of Immunology*	833	5.422
10	*Lancet*	810	79.321

IF, impact factors in 2020

**Fig. 2:**
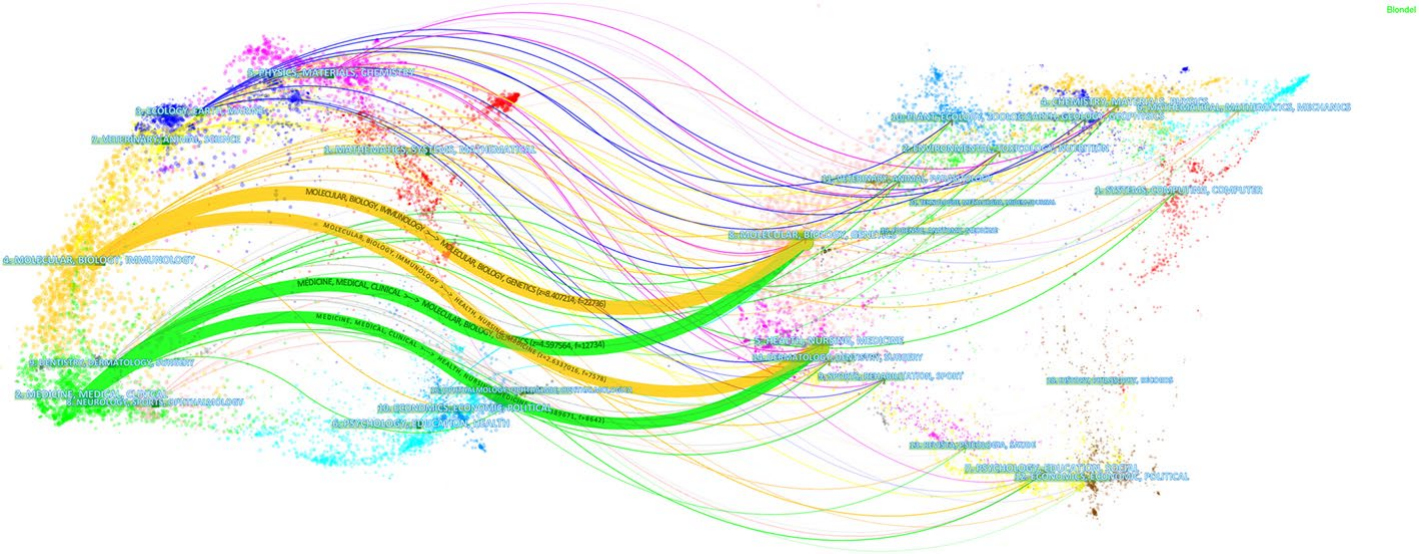
The dual-map overlay of gut–lung axis research. The dual-map overlay of journals represents the subject distribution of journals, with the left side of the graph representing citing journals and the right cited journals. The colored lines represent the citation relationship between articles in citing and in cited journals

A dual-map overlay graph of journals is shown in Fig. 2 to clarify the relationship between journals [17]. There are four main citation paths, two orange and two green. The orange paths indicate that the articles published in Molecular/Biology/Immunology journals often cite what was published in Molecular/Biology/Genetic and Health/Nursing/Medicine. The green paths indicate the articles published in Medicine/Medical/Clinical cite articles published in Molecular/Biology/Genetics and Health/Nursing/Medicine. The articles were published in the journals of medicine, health, molecule, gene, biology, immunity, nursing and other fields. All of the analysis above would provide a reference for the researchers in the field of the gut–lung axis.

### Country analysis

**Fig. 3:**
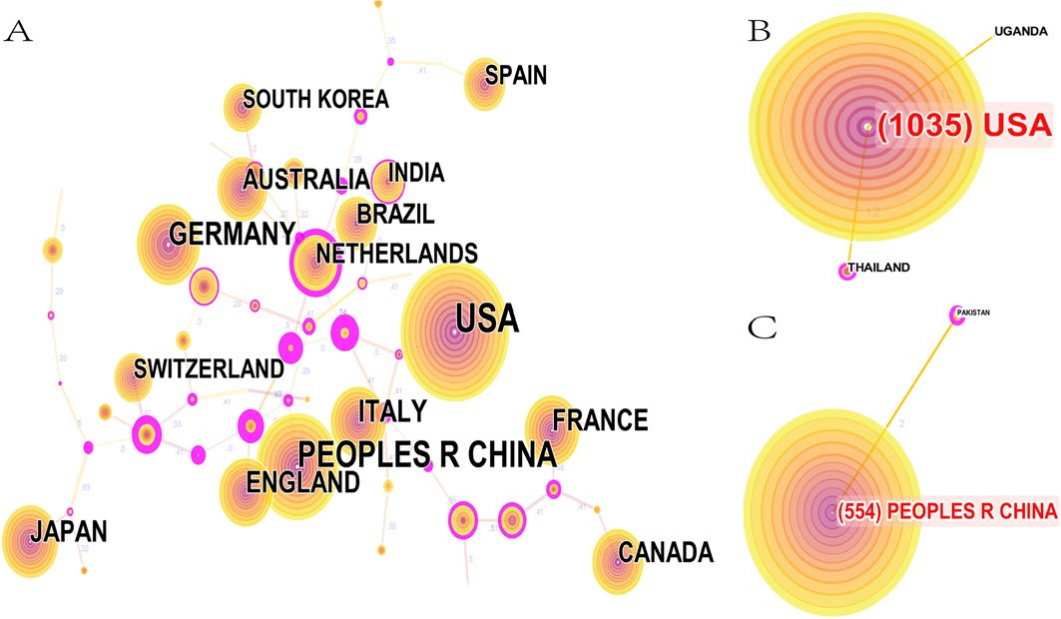
Co-occurrence analysis of countries. **A** Country co-occurrence map. **B** Cooperation network of the United States. **C** Cooperation network of China. The size of the node represents the number of publications. The link between nodes represents the existence of cooperation. The thickness of the lines represents the closeness of cooperation, and the thicker the lines are, the closer the cooperation is

The top 10 countries are listed in Table 4. The United States published the most articles (1035 articles), which accounted for nearly 1/3 of the total amount and surpassed China (554 articles) and Germany (255 articles). The country co-occurrence map is shown in Fig. 3A with 59 nodes and 65 links. It could be seen from Fig. 3B and C that the main cooperation countries of the United States were Thailand and Uganda in this field with the link strength of 0.12. The main cooperation country of China was Pakistan with a link strength of 0.2. The United States and China were the main research forces, however, their cooperation was not close in this field.

**Table 4:** Top 10 most publication countries

Ranking	Country	Frequency
1	United States of America	1035
2	The People's Republic of China	554
3	The Federal Republic of Germany	255
4	The Republic of Italy	203
5	Japan	190
6	The United Kingdom of Great Britain and Northern Ireland	176
7	The Republic of France	165
8	Commonwealth of Australia	153
9	Canada	147
10	The Federative Republic of Brazil	123

### Institution analysis

**Fig. 4:**
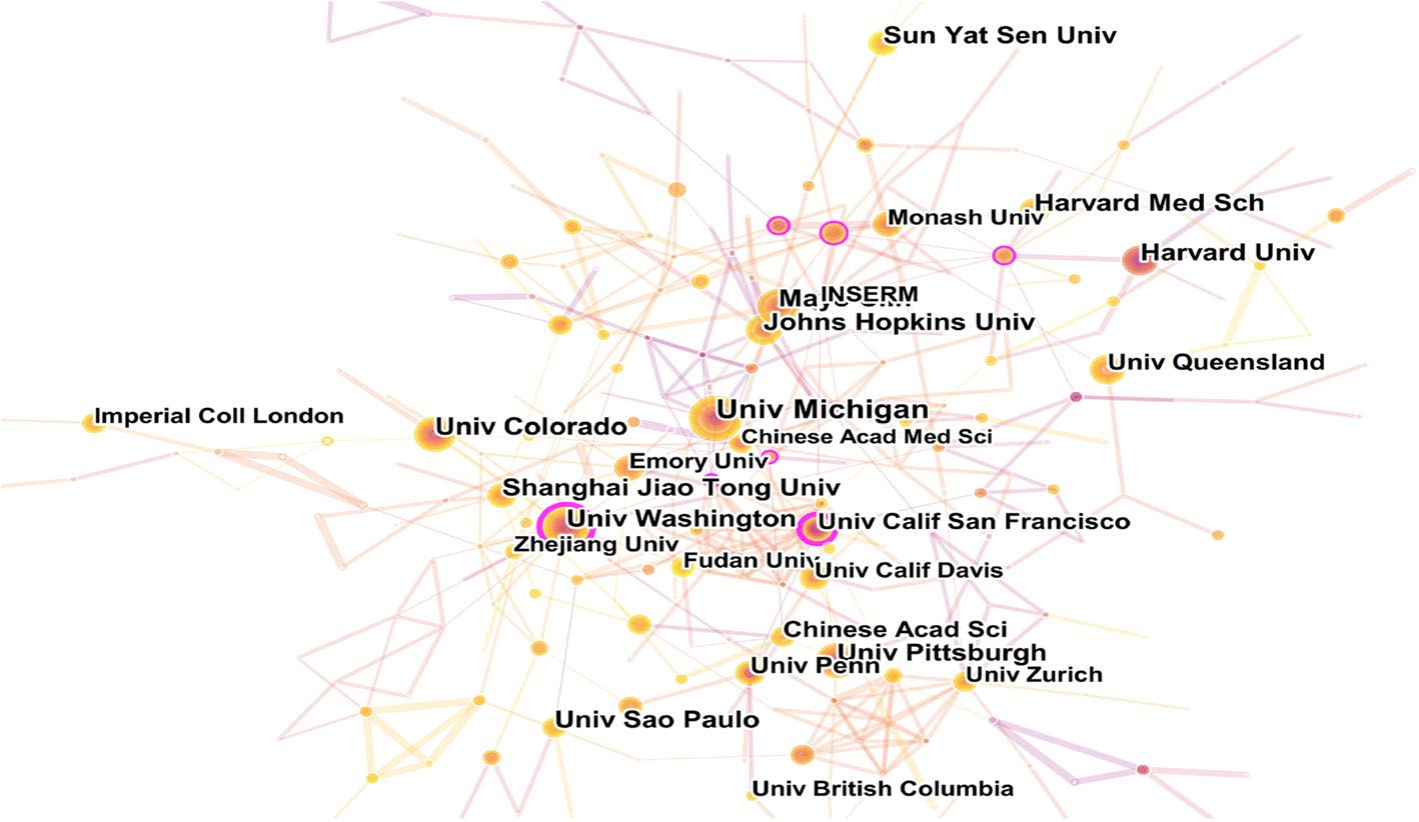
Co-occurrence analysis of institutions. The size of the node represents the number of publications, the link between nodes represents the cooperation between institutions, and the thickness of the lines represents the degree of cooperation

The institution co-occurrence map is shown in Fig. 4 with 357 nodes and 443 links. The top 10 institutions in the number of publications in this field are listed in Table 5. The University of Michigan was the most productive one (48 articles), followed by the University of Washington (32 articles) and Colorado State University (32 articles). Most are located in the United States among the top 10 institutions, and the support of American institutions has been an important factor for the dominance of the United States in this field.

**Table 5:** Top 10 most publication institutions

Ranking	Institution	Country/region	Frequency
1	The University of Michigan	United States	48
2	University of Washington	United States	32
3	Colorado State University	United States	32
4	Shanghai Jiao Tong University	China	30
5	Harvard University	United States	30
6	Johns Hopkins University	United States	30
7	Mayo Clinic	United States	30
8	Harvard Medical School	United States	29
9	Sun Yat-Sen University	China	29
10	University of Pittsburgh	United States	29

### Author analysis

**Fig. 5:**
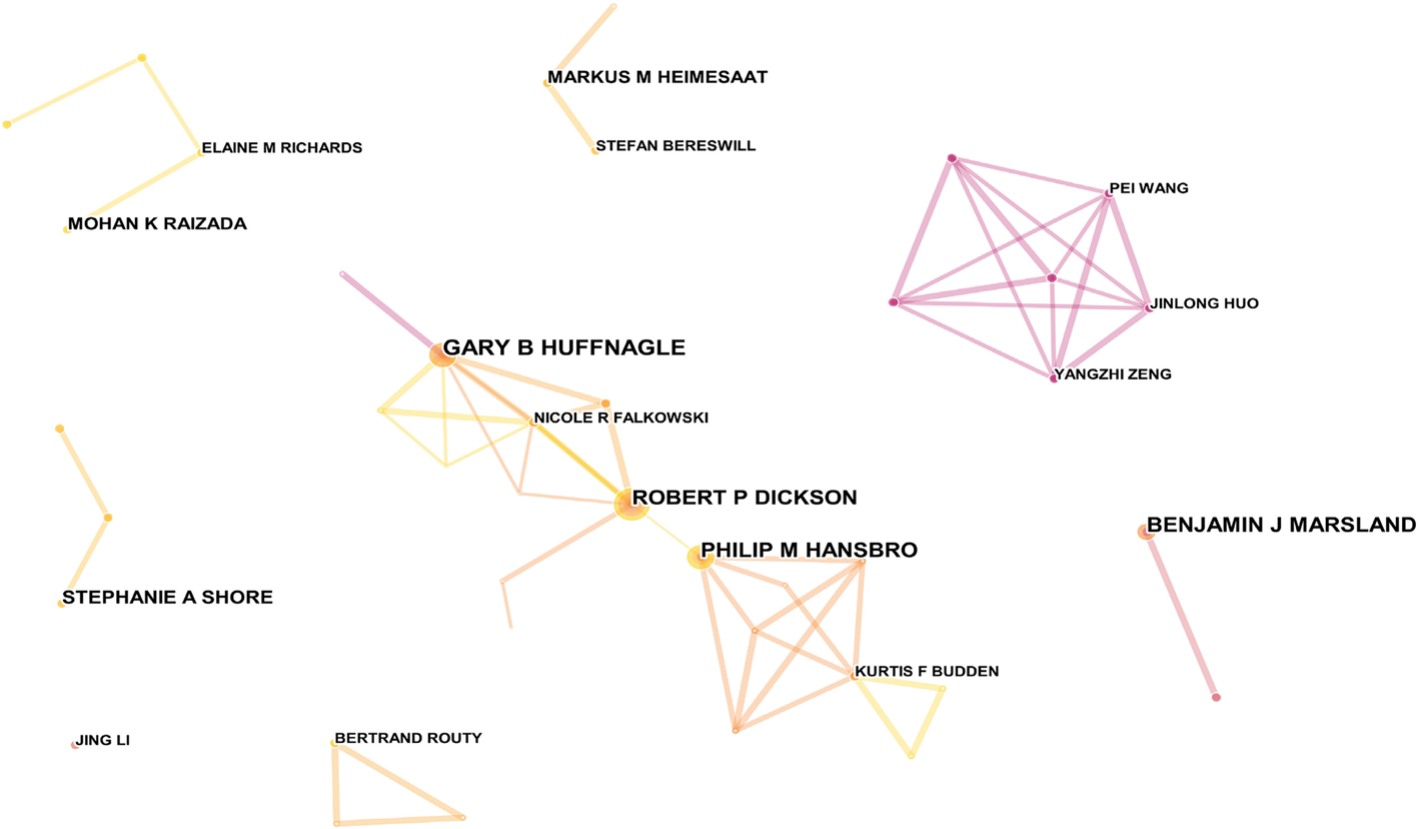
Co-occurrence analysis of authors. The size of the node represents the number of publications, the link between nodes represents the cooperation between authors and the thickness of the link represents the degree of cooperation

The author co-occurrence map is shown in Fig. 5, with 430 nodes and 658 links. The top 5 most productive authors and their affiliated institutions are shown in Table 6. The top 3 authors, Huffnagle Gary B, Dickson Robert P and Hansbro Philip M, have formed the largest cooperative network among many small scattered research groups. Dickson Robert P and Huffnagel Gary B were both from the University of Michigan Medical School.

**Table 6:** Top 5 most publication authors

Ranking	Frequency	Author	Country	Institution
1	13	Huffnagle Gary B	United States	University of Michigan Medical School
2	12	Dickson Robert P	United States	University of Michigan Medical School
3	11	Hansbro Philip M	Australia	The University of Newcastle
4	11	Marsland Benjamin J	Australia	Central Clinical School, Monash University
5	8	Shore Stephanie A	United States	Harvard University

### Keyword analysis

**Fig. 6:**
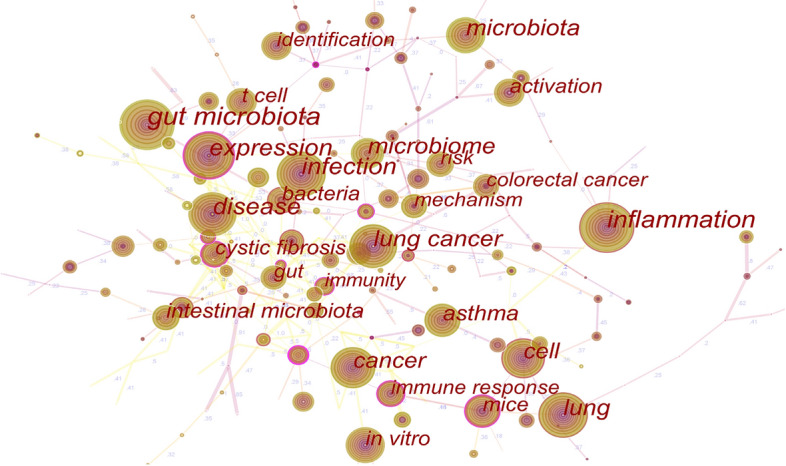
Co-occurrence analysis of keywords. The node size represents the number of occurrences. The larger the nodes are, the more frequent the keyword occurs. The co-occurrence keywords are linked with the lines, their thickness represents the degree of connection

The top 40 keywords with the most occurrences are shown in Table 7. It is shown that the most frequent keywords were “gut microbiota” (362), “inflammation” (280), “disease” (239) and “infection” (238). The keyword co-occurrence map is shown in Fig. 6 with 197 nodes and 283 lines. It is shown in Fig. 6 that “gut microbiota” often occurs together with “bacteria”, “supplement” and “pathology”. “Inflammation” often occurs together with “intestinal barrier”, “lung microbiome”, “tissue”, “epithelial cell” and “lipopolysaccharide”. “Infection” often occurs together with “diversity”, “Escherichia Coli” and “epidemiology”. “Disease” often occurs together with “protection”, “metabolite”, “expression”, “allergic asthma” and “exposure”.

**Table 7:** Top 40 keywords with the highest frequency of occurrence

Ranking	Frequency	Year of first occurrence	Keyword
1	362	2013	Gut microbiota
2	280	2011	Inflammation
3	239	2011	Disease
4	238	2011	Infection
5	226	2011	Expression
6	201	2013	Microbiota
7	195	2011	Lung
8	190	2014	Microbiome
9	179	2011	Lung cancer
10	167	2011	Cancer
11	154	2011	Cell
12	144	2011	Asthma
13	115	2015	Intestinal microbiota
14	106	2013	Bacteria
15	106	2011	Mice
16	105	2011	In vitro
17	100	2013	Cystic fibrosis
18	100	2012	Activation
19	99	2013	T cell
20	88	2011	Mechanism
21	86	2011	Colorectal cancer
22	85	2013	Risk
23	84	2011	Identification
24	83	2012	Gut
25	78	2011	Immune response
26	74	2015	Immunity
27	73	2011	Pathogenesis
28	73	2011	Diagnosis
29	72	2013	Diversity
30	70	2011	Model
31	67	2013	Probiotics
32	65	2011	Oxidative stress
33	62	2013	Cell lung cancer
34	62	2014	Children
35	58	2016	Health
36	58	2018	Immunotherapy
37	56	2015	Biomarker
38	55	2011	Therapy
39	54	2013	Metabolism
40	53	2011	Obstructive Pulmonary disease

**Fig. 7:**
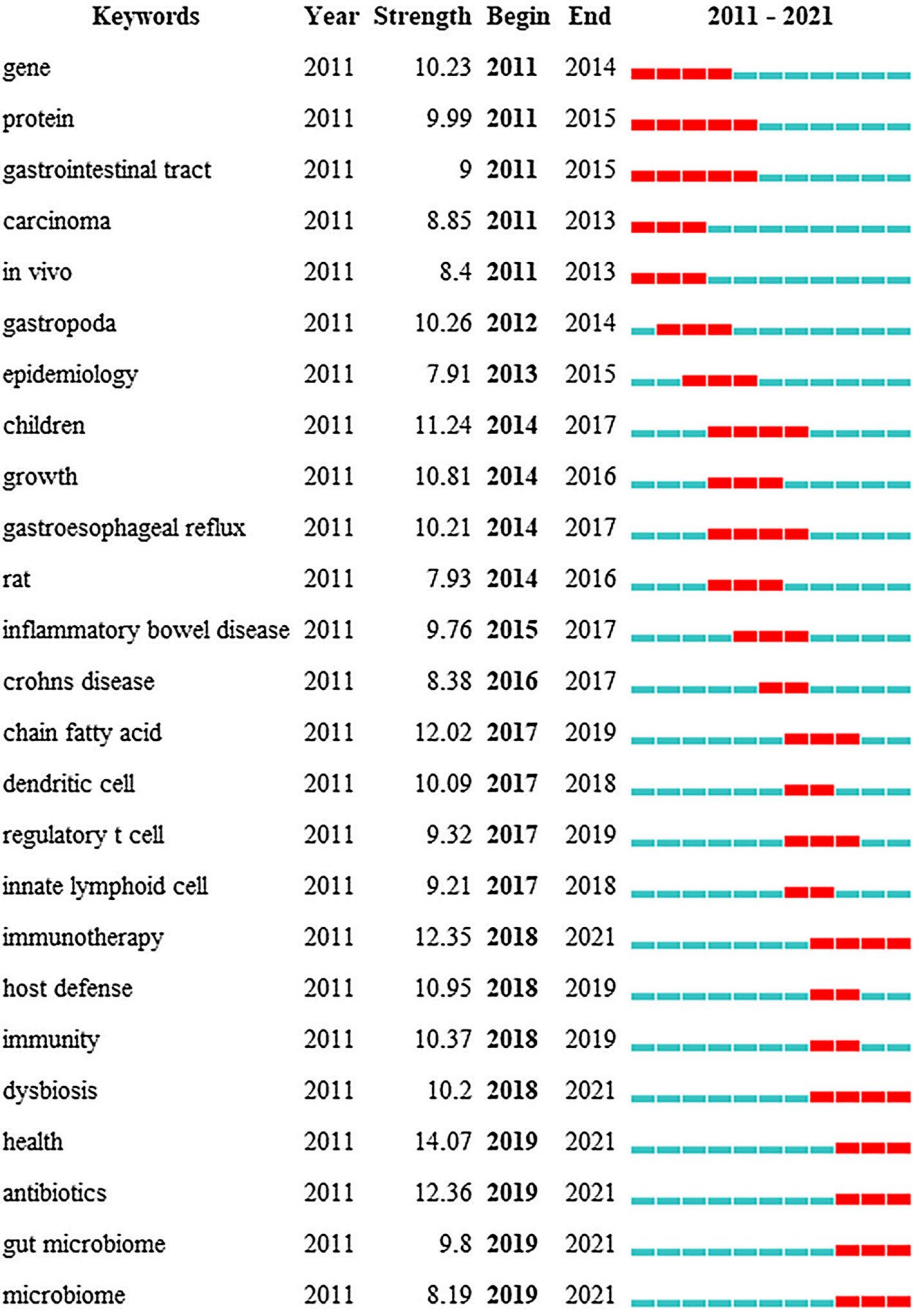
Top 25 keywords in burst impact. The blue and white squares in each row on the right side of the figure correspond to the year of hotspot. Red squares represent the year of hotspot, and blue squares represent non-hotspot year. The recent successive red squares represent the research hotspots in recent years

The top 25 keywords with burst impact are shown in Fig. 7. “Gene”, “protein”, “gastrointestinal tract”, “carcinoma”, “in vivo” and “gastropoda” were the keywords that had the earliest burst impact. “Epidemiology”, “children”, “growth”, “gastroesophageal reference”, “rat”, “inflammatory bowel disease” and “Crohn’s disease” had burst impact during 2014–2016. From 2017 to 2019, the keywords with burst impact were “chain fast acid”, “dental cell”, “regulatory T cell”, “internal lymphoid cell”, “immunity”, “host defense”, “immunity” and “dysbiosis.” The bursts with the most recent onset were “immunity”, “dysbiosis”, “health”, “antibiotics”, “gut microbiome” and “microbiome”, which indicated the forefront in the field of the gut–lung axis. The keyword with the highest strength was “health”, with a score of 14.07, followed by “antibiotics” and “immunotherapy”, with the score of 12.36 and 12.35, respectively.

### References analysis

**Fig. 8:**
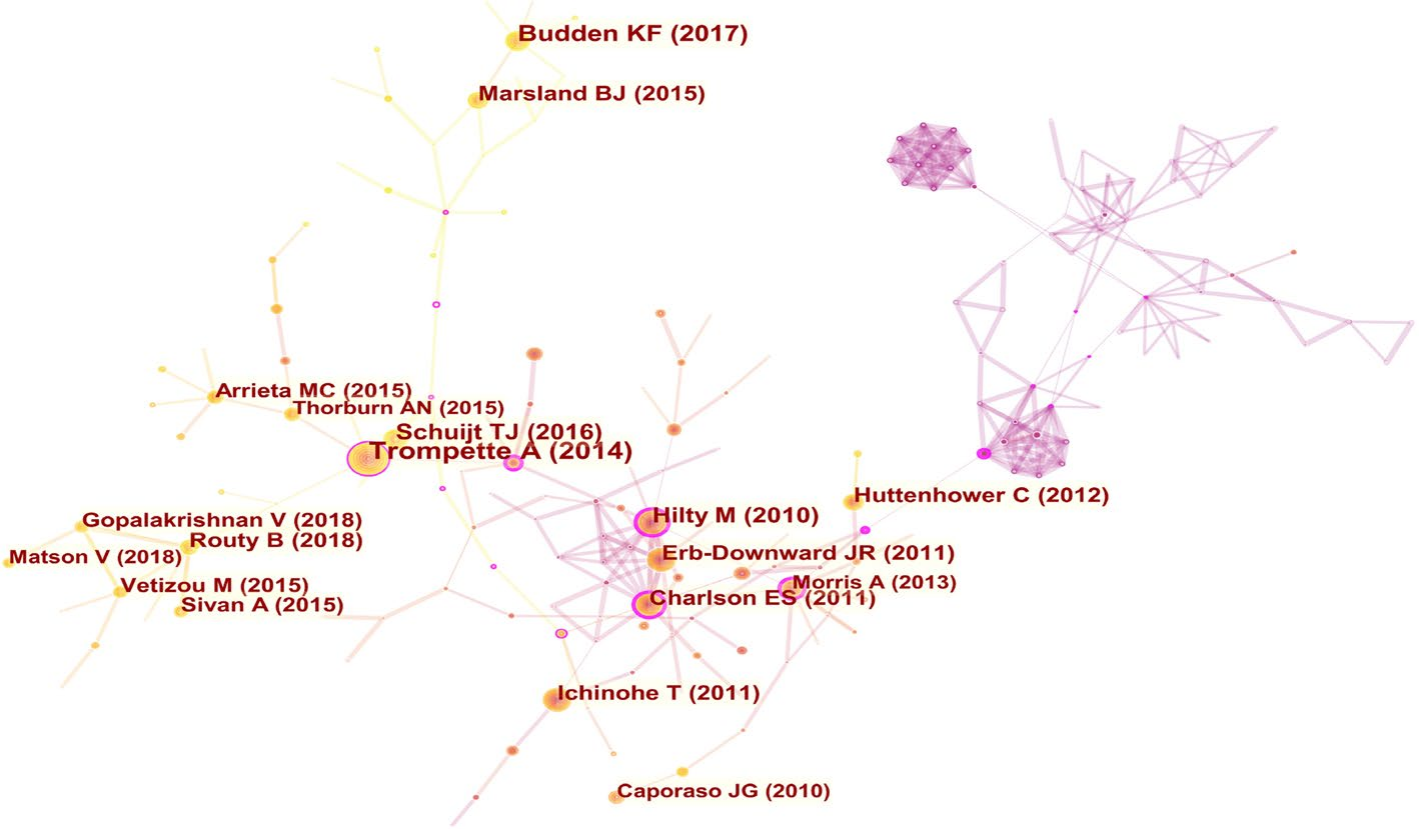
Co-occurrence analysis of references. The node size represents the citation frequency of the cited references, and the node with purple circle represents the key references. The larger purple circle indicates that the reference is more important

**Fig. 9:**
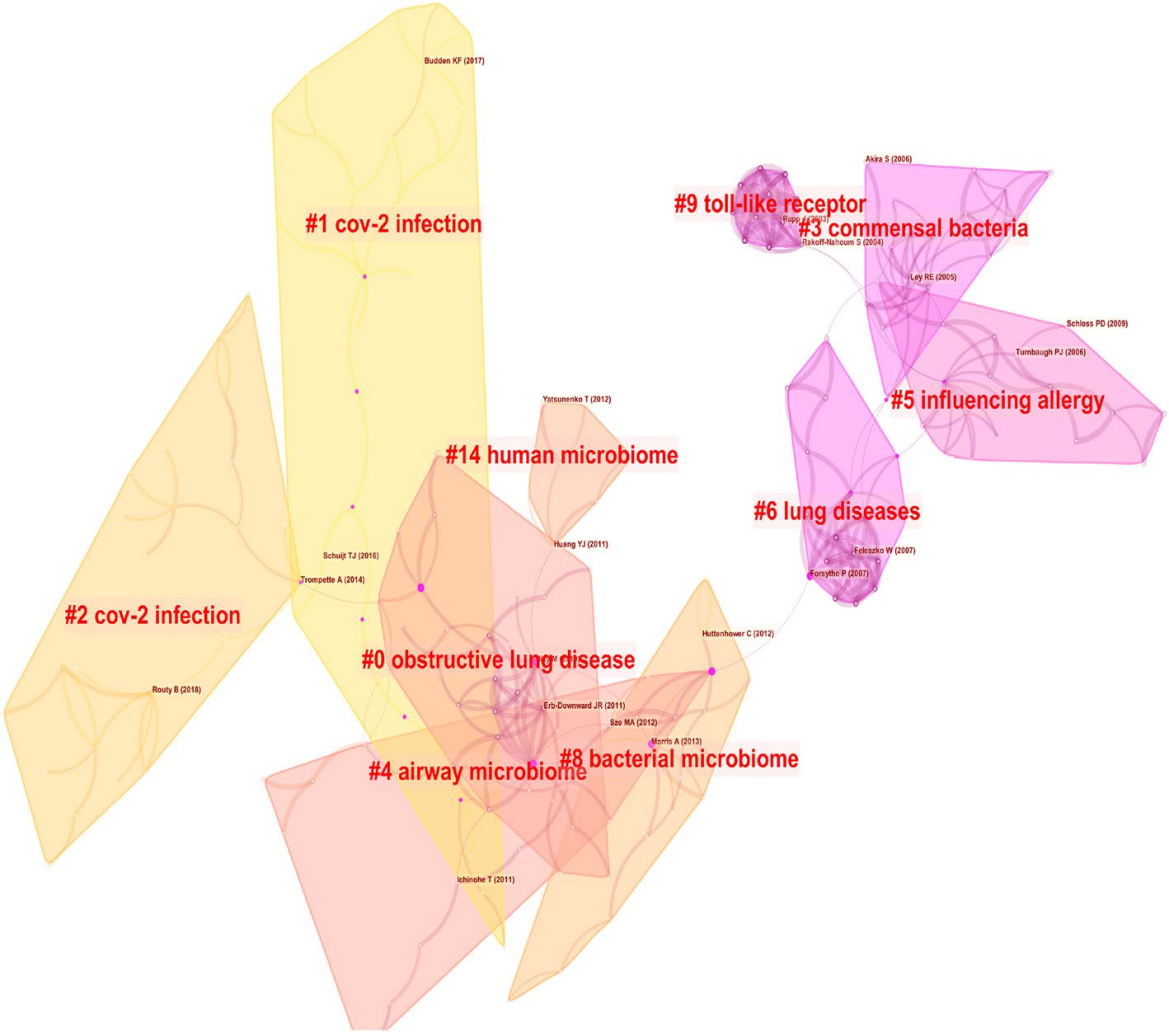
The cluster map of co-cited references. The cited references are clustered, each clustered box represents a category

**Fig. 10:**
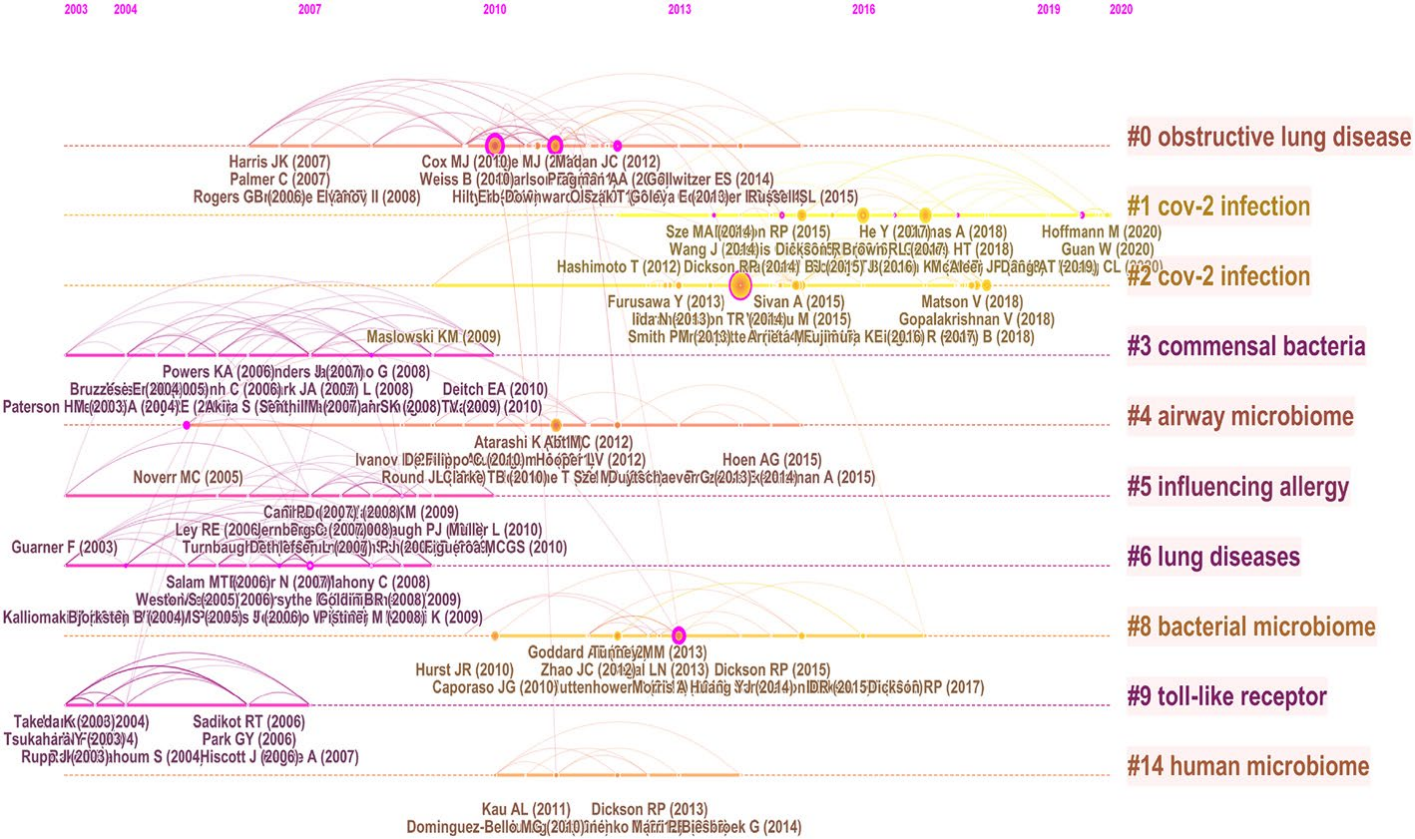
Timeline map of clustering of co-cited references. The results of clusters are shown at the right side, the warm color indicates a more recent cluster, and the cold color indicates an earlier one

The knowledge map of the co-occurrence references is shown in Fig. 8 with 313 nodes and 632 links. The nine largest clusters were presented by cluster analysis (Fig. 9), including #0 obstructive lung disease, #1 cov-2 infection, #2 cov-2 infection, #3 commensal bacteria, #4 airway microbiome, #5 influencing allergy, #6 lung diseases, #9 toll-like receptor, #14 human microbiome. It is shown in Fig. 10 that cov-2 infection has attracted much attention in recent years.

The top 10 cited references in this field are listed in Tables 8 and 9, which could be often considered fundamental in gut–lung axis research. The articles published by Trompette A in *Nature Medicine* had the highest number of citations (191), and the articles published by Hilty M had the highest centrality of score of 0.35. High centrality is often regarded as turning points or key points in a field [18].

**Table 8:** Top 10 most cited references

Authors	Frequency	Year of publication	Journal	Title	Focus
Trompette A [19]	191	2014	*Nature Medicine*	Gut microbiota metabolism of dietary fiber influences allergic airway disease and hematopoiesis	Fiber diet, bacterial metabolites, allergic airway disease
Budden KF [20]	122	2017	*Nature Reviews Microbiology*	Emerging pathogenic links between microbiota and the gut–lung axis	Gut–lung axis
Hilty M [21]	101	2010	*Plos One*	Disordered microbial communities in asthmatic airways	Dysbacteriosis of respiratory tract, asthmatic airways
Tim J Schuijt [22]	94	2016	*Gut*	The gut microbiota plays a protective role in the host defence against pneumococcal pneumonia	Bacterial pneumonia
Erb-Downward JR [23]	88	2011	*Plos One*	Analysis of the lung microbiome in the “healthy” smoker and in COPD	Pulmonary microorganism, chronic obstructive pulmonary disease
Emily S Charlson [24]	86	2011	*American Journal of Respiratory and Critical Care Medicine*	Topographical continuity of bacterial populations in the healthy human respiratory tract	Distribution of respiratory tract microbiome
Takeshi Ichinohe [25]	86	2011	*Proceedings of the national academy of sciences of the united states of America*	Microbiota regulates immune defense against respiratory tract influenza A virus infection	Immunity after influenza virus infection
Bertrand Routy [26]	84	2018	*Science*	Gut microbiome influences efficacy of PD-1-based immunotherapy against epithelial tumors	Intestinal flora, immune checkpoint inhibitors
Human Microbiome Project Consortium [27]	82	2012	*Nature*	Structure, function and diversity of the healthy human microbiome	Structure, function and diversity of microbiome
Benjamin J Marsland [28]	77	2015	*Annals of the American Thoracic Society*	The Gut–Lung Axis in Respiratory Disease	Intestinal flora, Respiratory diseases

**Table 9:** Top 10 references ranked by centrality

Author	Frequency	Year of publication	Journal	Title	Focus
Hilty M [21]	0.35	2010	*Plos One*	Disordered microbial communities in asthmatic airways	Dysbacteriosis of respiratory tract, asthmatic airways
Mairi C Noverr [29]	0.34	2005	*Infection and Immunity*	Development of allergic airway disease in mice following antibiotic therapy and fungal microbiota increase: role of host genetics, antigen, and interleukin-13	Antibiotic therapy, allergic airway, interleukin-13
Alison Morris [30]	0.32	2013	*American Journal of Respiratory and Critical Care Medicine*	Comparison of the respiratory microbiome in healthy nonsmokers and smokers	Differences of respiratory tract microbiome
Paul Forsythe [31]	0.32	2007	*American Journal of Respiratory and Critical Care Medicine*	Oral Treatment with Live Lactobacillus reuteri Inhibits the Allergic Airway Response in Mice	Probiotics, allergic airway
Emily S Charlson [24]	0.22	2011	*American Journal of Respiratory and Critical Care Medicine*	Topographical continuity of bacterial populations in the healthy human respiratory tract	Distribution of microorganisms in lung
Torsten Olszak [32]	0.21	2012	*Science*	Microbial exposure during early life has persistent effects on natural killer T cell function	Microbial exposure during early life, natural killer T cells
Christine M Bassis [33]	0.18	2015	*mBio*	Analysis of the upper respiratory tract microbiotas as the source of the lung and gastric microbiotas in healthy individuals	Source of respiratory tract microorganism
Trompette A [19]	0.17	2014	*Nature Medicine*	Gut microbiota metabolism of dietary fiber influences allergic airway disease and hematopoiesis	Fiber diet, bacterial metabolites, allergic airway
Jian Wang [34]	016	2014	*Journal of Experimental Medicine*	Respiratory influenza virus infection induces intestinal immune injury via microbiota-mediated Th17 cell-dependent inflammation	Influenza virus infection, Th17, intestinal immune injury
Rebecca L Brown [35]	0.15	2017	Rebecca L Brown	The microbiota protects against respiratory infection via GM-CSF signaling	Microbiota, GM-CSF signal, respiratory tract infection

**Fig. 11:**
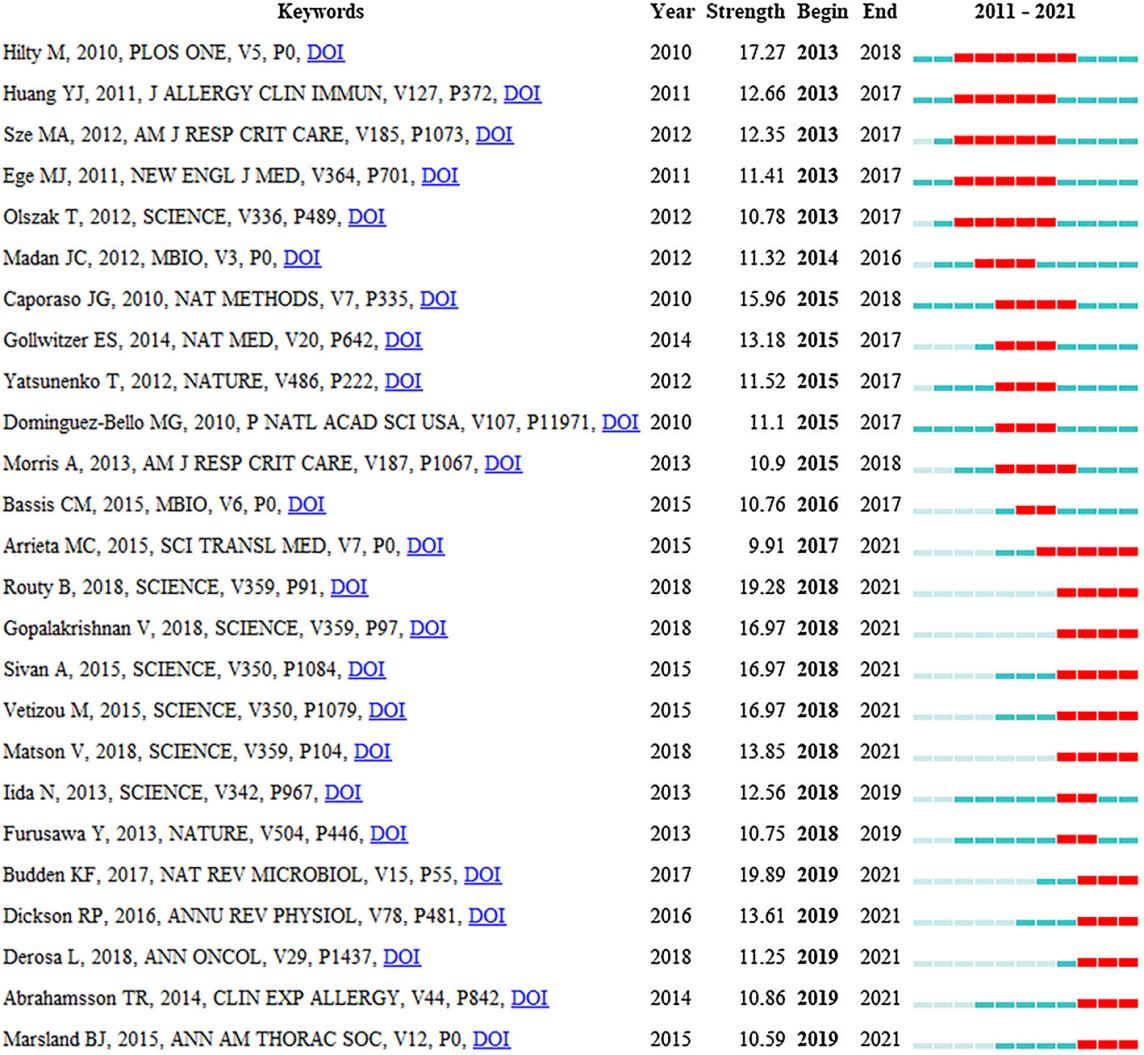
Top 25 cited references with burst impact. The blue and white squares in each row on the right side of the figure correspond to the year. The red squares represent that the references were highly cited in a short period and the recent successive red squares represent that the references were highly cited in recent years

Twenty-five references with burst impact are shown in Fig. 11. Eleven articles have been highly cited in recent 4 years, and 5 of them were published in the journal *Science* [26, 36–39], which were about the significance of microbiota in tumor immunotherapy. Researchers have found that programmed cell death protein-1 (PD-1) and programmed cell death protein ligand-1 (PD-L1) have significantly improved the survival rate of non-small cell lung cancer (NSCLC) [40], while antibiotic therapy led to an imbalance of intestinal flora, which affected the anti-tumor efficacy of immune checkpoint inhibitors (ICI). However the therapeutic effect of ICI was restored after manipulating the microbiota [26, 37, 38]. The studies above have provided a reference for clinicians in the application of ICI. The intervention of intestinal flora in improving the efficacy of lung cancer immunotherapy was a new frontier hotspot, which was consistent with the results of keyword burst detection.

## Discussion

Increasing attention has been paid to the field of gut–lung axis in recent years. Multiple intestinal diseases would result in respiratory symptoms and changes in the respiratory flora. Intestinal microbiota disorders are also present in patients with respiratory diseases. The antibiotic application may lead to disturbances in intestinal flora.

This bibliometric study aims to analyze the current status and trend of articles in the field of gut–lung axis in recent 10 years by using CiteSpace 5.7.R2. The results have demonstrated the important authors, core teams, active journals, research focuses and research development trends in this field.

The number of publications in this field has been increasing, which indicates a broad research prospect in the field of the gut–lung axis. The top 10 journals accounted for 1/9 of the total studies, suggesting that the articles were widely distributed among various journals. The greatest number of articles on the gut–lung axis were published on *Plos One* and were cited the most frequently. As the most active journal in this field, *Plos One* has a certain influence in the field of the gut–lung axis.

The analysis of the collaboration network map has indicated that the United States was the leading research force in the field of the gut–lung axis. Besides the high participation of American Institutions, the large amount of the United States’ financial support for the field may be another important factor for the United States to be dominant in this field. Although the United States and China have produced the largest number of research articles, their cooperation is not close and strengthening the cooperation between which in this field may contribute to more ground-breaking results. Among the three key authors in this field, both Dickson Robert P and Huffnagle Gary B came from the University of Michigan Medical School who focused on the field of respiratory and critical medicine. Researchers have found that the lung microbiome is rich in gut-associated bacteria in patients with sepsis and acute respiratory distress syndrome (ARDS) [41]. The key characteristics of the lung microbiome (bacterial load and enrichment of gut-associated bacteria) were correlated with ARDS, which could predict the prognosis of critical patients [42, 43]. The hyperoxia would cause changes in the microbiota of the lung and intestines and would lead to lung injury [44]. Hansbro Philip M has studied widely and achieved some research results in the effect of diet on mucosal immunity [45], short-chain fatty acids (SCFAs), and the role of inflammasomes in regulating intestinal and pulmonary inflammation [46, 47], and 146 bacterial species were found to differ between the patients with COPD and normal individuals by examining the fecal microbiome [48].

Keyword analysis has identified 3 research focuses in the field of the gut–lung axis: *Inflammation*, *Infection* and *Disease*. (1) *Inflammation:* The gut flora and its metabolites are critical in maintaining normal mucosal immune function [49]. The mucosal barrier is rich in Group 2 initial lymphocytes that can migrate from the gut to the lung to participate in the inflammatory process [50, 51]. Respiratory immune response belongs to the category of the mucosal immune response. The commensal microflora would contribute to activating human immune cells after bacteriae, viruses or other pathogenic microorganisms infection [22, 25, 52, 53]. In addition, SCFAs, the metabolites of the intestinal flora, plays a significant role in preventing airway allergic reaction and inhibiting airway inflammation [19, 54, 55]. (2) *Infection*: When there is pulmonary bacterial infection, the intestinal flora would increase host defense through toll-like receptor and nod-like receptor signaling [52, 56]. After influenza A virus infection, the intestinal flora is involved in the activation of inflammasomes, contributing to dendritic cell migration [25]. Moreover, Bifidobacterium could regulate Th1/Th2 immune response and enhance the disease resistance of mice [57]. (3) *Disease*: The three most related ones are pulmonary obstructive disease, cystic fibrosis and lung cancer. ① The occurrence of asthma is closely related to the early intestinal flora imbalance in children, which is associated with early life antibiotic exposure and the severity of asthma has a dose-dependent correlation with antibiotics [58–61]. Appropriate probiotic supplementation was beneficial to the treatment of asthma [56, 62]. Additionally, patients with obstructive pulmonary disease had a higher risk of IBD and IBS [63, 64]. Moreover, IBD also increases the mortality of patients with COPD and asthma [65]. ② Cystic fibrosis is an autosomal recessive genetic disease, mainly characterized by respiratory and gastrointestinal symptoms, which reflects the correlation between lung and gut. Compared with healthy subjects, Faecalibacterium, Roseburia and Bifidobacterium decreased in the intestinal tract in patients with cystic fibrosis. However, breastfeeding or probiotic application was beneficial to the recovery of intestinal flora structure, which could reduce the deterioration of the pulmonary condition and the number of hospitalizations [66, 67]. ③ The abundance of Firmicutes and Proteobacteria is relatively low in patients with lung cancer while relatively high in Bacteroidetes and Fusobacteria [68]. Lipopolysaccharide produced by G-bacilli in the gut could induce inflammatory response and lung metastasis of melanoma, while the changes in the intestinal flora could prevent this process [69]. Furthermore, diversities of intestinal flora are also crucial in the immunotherapy in lung cancer [26, 37, 38, 70].

The references analysis has revealed the important references in the field of the gut–lung axis in the past 10 years. The references listed in Tables 8 and 9 would provide an important reference for the study in this field. Additionally, the timeline of references analysis (Fig. 8C) suggests that CoV-2 infection has attracted much attention in this field in recent years. Studies have found that intestinal flora imbalance could lead to the destruction of the intestinal barrier, which may contribute to SARS-CoV-2 transferring from the lung to the intestines through the circulatory and lymphatic system, leading to secondary infection and multiple organ failure [53, 71, 72]. The use of probiotics could significantly improve fever, cough, diarrhea and other clinical symptoms of COVID-19 patients and reduce the risk of respiratory failure [73, 74], which provides a new direction for the treatment of COVID-19. Moreover, burst detection demonstrates that immunotherapy, antibiotics, dysbiosis, health, gut microbiome and microbiome are new research directions in the field of the gut–lung axis. Antibiotics could lead to dysbacteriosis, affecting the efficacy of tumor immunotherapy. It is a frontier field of the improvement of immunotherapy efficacy in lung cancer by modulating intestinal flora.

## Conclusion

Based on the results of CiteSpace, this study has identified the important journals, countries and collaborators in the field of the gut–lung axis. According to the keywords, references and burst detection, the research focuses and frontier hotspots of the gut–lung axis were determined. In addition, new therapeutic targets in gut microbiota have great potential in treating pulmonary diseases.

This study retrieved publications from the WoSCC with the limitation of language (English) and literature type (article and review), which may not be sufficient in the representation of all the current research on the gut–lung axis. However, this study has covered the majority of articles in the field of the gut–lung axis in recent 10 years, which could reflect the overall status and trends in this field.

## Methods

### Search strategy

WoSCC has a wide range of selective literature and its data analysis format meets the requirements of CiteSpace software. We reviewed papers published in the past 10 years on WoSCC on March 26, 2021. The retrieval strategies are as follows:

$$ \left( { \, \left( {{\text{TS}} = \left( {{\text{gastro}}*{\text{ micro}}*} \right){\text{ OR TS}} = \left( {{\text{gastro}}*{\text{ flora}}} \right){\text{ OR TS}} = \left( {{\text{gut micro}}*} \right){\text{ OR TS}} = \left( {\text{gut flora}} \right){\text{ OR TS }} = \, \left( {{\text{intestin}}*{\text{ micro}}*} \right){\text{ OR TS }} = \, \left( {{\text{intestin}}* {\text{flora}}} \right) \, } \right){\text{ and }}\left( {{\text{TS }} = \, \left( {{\text{lung}}*} \right){\text{ OR TS }} = \, \left( {{\text{pulmo}}*} \right) \, } \right) \, } \right){\text{ OR TS }} = \, \left( {{\text{gut}}{-}{\text{lung axis}}} \right) $$

The language was “English”, the document types included “article” and “review”. “Procedures paper”, “book chapter”, “data paper”, “early access” and “retracted publication” were excluded; publication time was from 2011/01/01 to 2021/3/26. 3315 articles (including 2469 articles and 846 reviews) were screened out. The “fully recorded and cited references” of these documents were extracted into CiteSpace 5.7.R2 in “plain text” format to identify the main countries, institutions, authors, keywords and references.

### Parameter settings

The parameters of CiteSpace 5.7.R2 were set as follows:

1. Time slicing: each year as a time slice from 2011 to 2021.
2. Term source: title, abstract, author keywords, and keywords plus.
3. Node types: author, institution, country, keywords, reference.
4. Top Nperslice: “Top Nperslice = 50” for author, institution, country and keyword node type, “Top Nperslice = 25” for reference node type.
5. Pruning options: Pathfinder, pruning the merged network. The information of country, author, institution, keywords and references were analyzed visually.

## Data Availability

All the data used to support the findings of this study are available from the corresponding author upon reasonable request.

## Abbreviations

COPD: Chronic obstructive pulmonary disease
IBD: Intestinal bowel disease
IBS: Intestinal bowel syndrome
LPS: Lipopolysaccharide
WoSCC: Web of Science Core Collection
PD-L1: Programmed cell death protein ligand-1
PD-1: Programmed cell death protein-1
ICI: Immune checkpoint inhibitors
SCFAs: Short-chain fatty acids
ARDS: Acute respiratory distress syndrome
